# Does Maintained Medical Aid Coverage Affect Healthy Lifestyle Factors, Metabolic Syndrome-Related Health Status, and Individuals’ Use of Healthcare Services?

**DOI:** 10.3390/healthcare11131811

**Published:** 2023-06-21

**Authors:** Ilsu Park, Kyounga Lee, Eunshil Yim

**Affiliations:** 1 Department of Healthcare Management, Dong-eui University, 176 Eomgwang-ro, Busanjin-gu, Busan 47340, Republic of Korea; ispark@deu.ac.kr; 2 College of Nursing, Gachon University, 191 Hambangmoe-ro, Yeonsu-gu, Incheon 21936, Republic of Korea; 3 Department of Nursing, Daegu Health College, 15 Yeongsong-ro, Buk-gu, Daegu 41453, Republic of Korea; yim7604@dhc.ac.kr

**Keywords:** medical aid, universal health coverage, healthy lifestyle, metabolic syndrome, healthcare utilization, moral hazards

## Abstract

Concerns about the moral hazards and usage of universal health insurance require examination. This study aimed to analyze changes in lifestyle, metabolic syndrome-related health status, and individuals’ tendency to use healthcare services according to changes in the eligibility status of medical aid recipients. This paper reports a retrospective cohort study that involved analyzing data from 2366 medical aid recipients aged 40 years or older who underwent national health screenings in 2012 and 2014. Of the recipients, 1606 participants continued to be eligible for medical aid (the “maintained” group) and 760 changed from being medical aid recipients to National Health Insurance (NHI) enrollees (the “changed” group). Compared to the “changed” group, the “maintained” group was less likely to quit smoking, more likely to begin smoking, less likely to reduce binge drinking to moderate drinking, and had a significant increase in blood glucose and waist circumference. Annual total medical expenses also increased significantly in the “maintained” group. Since the mere strengthening of healthcare coverage may lead to moral hazards and the failure to link individuals’ tendency to use healthcare services and outcomes, establishing mechanisms is necessary to educate people about the health-related outcomes of maintaining a healthy lifestyle and ensure the appropriate use of healthcare services.

## 1. Introduction

Providing universal health insurance for all is a global imperative [1]. Most European countries have a national health service to ensure that all citizens have access to the healthcare services they need [2,3], and the U.S. is developing and expanding the Affordable Care Act (ACA), Medicaid, Medicare, and other policies to achieve near-universal health insurance coverage [4,5]. South Korea also has universal healthcare coverage, which is divided into health insurance and medical aid [6]. The country has a National Health Insurance (NHI) system in which it is the single insurer, and all citizens are automatically enrolled in a single health insurance plan. All citizens with income above a certain amount pay a monthly health insurance premium, and they and their dependents can access the services provided by medical institutions after paying a certain amount of deductibles. For low-income citizens who are unable to support themselves or have difficulty earning a living, the government has a medical aid system that provides taxpayer-funded medical care and check-up services [7]. When medical aid recipients use healthcare facilities, they pay a co-payment that is either free or considerably low compared to that of NHI enrollees [6,8,9].

In 2022, the number of medical aid recipients in Korea was 1,522,292 (2.9%), and the number of NHI enrollees was 51,409,978 (97.1%). Furthermore, the total medical expenses used by medical aid recipients was KRW 10,334 billion (approximately USD 8 billion), of which 97.2%, or KRW 10,479 billion (approximately USD 7.7 billion), was paid by the government [10].

When recipients do not receive health checkups or preventive health care when they are healthy, they will require more healthcare services when they become ill [11,12]. Therefore, South Korea began providing general health screening services to medical aid recipients in 2012 [13].

While there is agreement on the need for universal health coverage, views on medical aid coverage vary. Studies have shown that medical aid recipients have lower levels of health and higher levels of unmet medical needs than NHI enrollees (or private health insurance enrollees) [6,14,15]. Since medical aid coverage can reduce unmet medical needs and improve health outcomes for vulnerable populations, expanding the coverage is necessary [6,14,15]. However, medical aid coverage has been found to increase the utilization of healthcare facilities, and if this exceeds an appropriate level, it can lead to the unnecessary use of healthcare services; therefore, controlling the use of healthcare services by carefully selecting recipients is necessary [9,16,17,18]. In many other countries, public insurance for low-income people raises concerns over moral hazards, and the excessive use of healthcare services places a strain on finances [19,20]. Moral hazards (also known as utilization risks) comprise a model based on behavioral economics that suggests that medical aid coverage reduces the health-related financial burden of illness by lowering healthcare costs; however, this tends to reduce preventive activities and leads to the unnecessary use of healthcare services [18]. The question of whether medical aid recipients make use of healthcare services more often due to their poorer health status or simply because there are fewer financial barriers to access is of ongoing concern [21]. As co-payments for healthcare services increase, people are less likely to make use of healthcare services [22], which may lead to efforts to enhance their health levels by improving healthy lifestyle behaviors [23]. Previous studies have demonstrated that NHI enrollees with lower coverage are less likely to make use of healthcare services than medical aid recipients [24].

It should be acknowledged that poor living conditions associated with low-income individuals negatively affects their healthy lifestyle choices and health status [7,25]. Since individuals with private insurance or health insurance are quite different from those reliant on medical aid, little evidence can be found on the comparative effects of public vs. private coverage [8,16]. In other words, rather than simply comparing medical aid recipients to health insurance recipients, making comparisons using similar groups would be more meaningful [9].

Drinking alcohol, smoking, and exercising are the main behaviors that affect one’s health [19,26]. Numerous studies have demonstrated the importance of avoiding smoking, controlling alcohol consumption, and exercising, all of which can help an individual maintain a healthy lifestyle [12,27,28]. An unhealthy lifestyle is significantly associated with the prevalence of metabolic syndrome [29]. Metabolic syndrome is a factor that greatly affects health, one’s tendency to use healthcare services, and healthcare costs [30,31]. Risk factors for metabolic syndrome, including blood pressure, blood sugar, waist circumference, triglycerides, and HDL levels, can be used to determine the risk of developing chronic diseases such as diabetes, hypertension, chronic kidney disease, and cardiovascular disease. Metabolic syndrome has been reportedly associated with the incidence and mortality of various cancers, such as breast and rectal cancer; therefore, diagnosing and treating metabolic syndrome is of great clinical importance, and the condition should be managed to reduce disease burden [32,33]. It is also a meaningful predictor of one’s tendency to use healthcare services because metabolic syndrome is a preventable risk factor and not something which should require emergency treatment [29]. Since healthy lifestyle and metabolic syndrome factors are also associated with low socio-economic status [29], paying attention to what makes a healthy lifestyle and the factors related to metabolic syndrome, specifically regarding medical aid recipients, are necessary.

Against this backdrop, determining whether the healthy lifestyles and metabolic syndrome-related health status of individuals differ depending on the maintenance of medical aid coverage is necessary. Additionally, analyzing the impact on individuals’ tendency to use healthcare services is necessary. To this end, groups with similar socio-economic characteristics need to be compared. In this study, we analyzed changes in lifestyle, metabolic syndrome-related health status, and medical expenditure among medical aid recipients who maintain eligibility for medical aid and those whose eligibility changed, forcing them to transition from being medical aid recipients to NHI enrollees.

## 2. Materials and Methods

### 2.1. Study Design

This paper presents a retrospective cohort study analyzing changes in lifestyle and metabolic syndrome-related health status, as well as individuals’ tendency to use healthcare services in those eligible for medical aid (the “maintained” group) and those who changed from being medical aid recipients to NHI enrollees (the “changed” group).

### 2.2. Study Sample

The sample comprised medical aid recipients aged 40 years or older who underwent national health screenings in 2012 and 2014. Of these, we analyzed the data of 2366 individuals for whom both healthy lifestyle and metabolic syndrome risk factor levels, the main variables of the study, were recorded. Between 2012 and 2014, 1606 subjects in the sample remained medical aid recipients, and 760 changed from being medical aid recipients to NHI enrollees.

### 2.3. Data Collection and Ethical Considerations

This study used national health screening and National Health Insurance data received from the Korea National Health Insurance Service (NHIS). The NHIS provided de-identified national health screening and healthcare utilization data for policy and academic research purposes. Since this study did not collect and record personal information and instead used information that has already been disclosed to the public, a review exemption was approved by the Institutional Review Board of Gachon University (IRB No. 1044396-202303-HR-046-01).

### 2.4. Study Measures

#### 2.4.1. Whether Medical Aid Eligibility Is Maintained: “Maintained” vs. “Changed”

Participants who were medical aid recipients in both 2012 and 2014 were categorized into the “maintained” group, and those who were medical aid recipients in 2012 but whose status changed to NHI enrollees in 2014 were categorized into the “changed” group. In Korea, the national health screening service is implemented every two years, and since it started in 2012 for medical aid recipients, qualifications in 2012 and 2014 were used. The “changed” group refers to a change in health insurance coverage (e.g., those who transition from being medical aid recipients in 2012 to NHI enrollees in 2014 for reasons such as income generation).

#### 2.4.2. Healthy Lifestyle

The healthy lifestyle factors analyzed in this study were smoking, exercise, and alcohol consumption. The criteria of previous similar studies were applied to derive the criteria for these factors [28]. In terms of smoking, individuals were categorized as smokers if they were current smokers at the time of the health screening and non-smokers if they were not. Regarding exercise, they were categorized into the physical activity (PA) group if they exercised or walked for at least 30 min a day, 5 days a week, while those who did not exercise were sorted into the non-PA group. Regarding alcohol consumption, men were categorized as binge drinkers if they drank more than seven glasses of any alcoholic beverage at least twice per week, while for women to be considered binge drinkers, this figure had to be five glasses or above. The remaining were classified as moderate drinkers.

#### 2.4.3. Metabolic Syndrome-Related Health Status

The health status of individuals was measured using five factors of metabolic syndrome (blood pressure, blood glucose, waist circumference, triglycerides, and HDL). The risk group classification criteria were based on the diagnostic criteria for metabolic syndrome as follows [33,34,35]. Individuals were classified as a risk group if their blood pressure was 130/85 mm Hg or higher or if they took antihypertensive medication; if fasting blood glucose was 100 mg/dL or higher or if they took glycemic control medication; if waist circumference was more than 85 cm for women and more than 90 cm for men; if triglycerides were more than 150 mg/dL; and if HDL was less than 40 mg/dL for men or less than 50 mg/dL for women. Those with three or more of these five criteria were classified as having metabolic syndrome.

#### 2.4.4. Healthcare Utilization

Healthcare utilization was measured using annual total medical expenses. Annual total medical expenses were considered to be individuals’ total medical expenditure, including national (health insurance) contributions and co-payments, per year.

### 2.5. Statistical Analysis

The general characteristics, healthy lifestyle factors, and health status of individuals were presented using means, standard deviations, frequencies, and percentages, and the differences between the “maintained” group and the “changed” group were analyzed by conducting χ^2^-tests and *t*-tests. Differences in the levels of five factors of metabolic syndrome and medical expenses between 2012 and 2014 were analyzed by conducting paired *t*-tests. Hierarchical logistic multiple regression analyses were conducted to determine the impact of maintaining eligibility for medical aid on whether total medical expenses increased. Model 1 was adjusted for demographic characteristics; Model 2 was adjusted for demographic characteristics and healthy lifestyle change characteristics (smoking, drinking, and exercise); and Model 3 was adjusted for demographic characteristics, healthy lifestyle change characteristics, and health status change characteristics (blood pressure, blood glucose, triglycerides, HDL, waist circumference). All data analyses were performed using SPSS 24 version (IBM SPSS Statistics, Armonk, NY, USA)

## 3. Results

### 3.1. Baseline Characteristics

Regarding those screened in 2012, there were 1599 women (67.6%) and 767 men (32.4%), with a mean age of 47.53 ± 7.42 years (Table 1). The number of non-smokers comprised 83.0% of the studied population, and moderate drinkers comprised 90.7% of the studied population. A total of 72.1% of the studied population were placed in the non-PA group. There were 1774 (25.0%) individuals with metabolic syndrome; 54.9% had a normal blood pressure, 60.9% had normal blood sugar levels, 72.5% had normal triglycerides, 73.2% had normal HDL, and 76.3% had a normal waist circumference. There were no differences in gender, smoking, exercise, HDL, or waist circumference between the maintained and changed groups, but there were differences in age, drinking, blood pressure, blood glucose, triglycerides, and the presence of metabolic syndrome (*p* < 0.01). Compared to the “maintained” group, the “changed” group was older and had higher rates of binge drinking, blood pressure, blood glucose, and triglyceride risk.

### 3.2. Changes in the Healthy Lifestyle and Metabolic Syndrome-Related Health Status

The changes in lifestyle factors and metabolic syndrome-related health status among the studied subjects are presented in Table 2. As for healthy lifestyle factors, there were differences in smoking (X^2^ = 8.033, *p* = 0.045) and drinking (X^2^ = 15.636, *p* = 0.001) between those who maintained the eligibility for medical aid and those who did not. As for health status, there were differences in blood pressure (X^2^ = 32.112, *p* < 0.001), blood glucose (X^2^ = 11.683, *p* = 0.009), triglycerides (X^2^ = 13.765, *p* = 0.003), and the presence of metabolic syndrome (X^2^ = 16.047, *p* = 0.001). The proportion of people who went from smoking to non-smoking was lower in the “maintained” than in the “changed” group (2.9% vs. 3.6%). This was also more than twice as high in the “maintained” than in the “changed” group (3.1% vs. 1.4%). As for drinking, the rate of transition among those going from being binge drinkers to moderate drinkers was more than twice as high in the “changed” group (3.5% vs. 7.1%). For blood pressure and blood glucose, the rate of change from being in the risk group to normal in both 2012 and 2014 was higher in the “changed” group, and for triglycerides, the rate of change from being in the risk group to normal was also higher in the “changed” group.

### 3.3. Differences in Metabolic Syndrome-Related Health Status and Medical Expenses

The “maintained” group had a significant increase in blood glucose (t = −3.495, *p* < 0.001) and waist circumference (t = −4.028, *p* < 0.001) compared to the “changed” group, while the systolic blood pressure (t = 2.154, *p* = 0.032) and triglycerides levels (t = 2.088, *p* = 0.037) statistically significantly decreased in the “changed” group (Table 3). Total annual medical expenses were statistically significantly increased in the “maintained” group (t = −3034, *p* = 0.002).

### 3.4. Relationship between Medical Aid Eligibility and Increase in Total Medical Expenses

The impact of changes in medical aid eligibility on the increase in total medical expenses is presented in Table 4. Those who remained eligible for medical aid (“maintained” group) were 1.368 times more likely to have an increase in total medical expenses compared to the “changed” group(OR = 1.368, 95% CI = 1.141–1.641) in Model 1 (adjusted for demographic characteristics—gender and age) and 1.400 times more likely in Model 2, adjusted for demographic characteristics and healthy lifestyle change characteristics (smoking, drinking, and exercise) (OR = 1.400, 95% CI = 1.165–1.683). In Model 3, the “maintained” group was 1.421 times more likely to experience an increase in total medical expenses than the “changed” group (OR = 1.421, 95% CI = 1.180–1.710), adjusted for demographic characteristics, healthy lifestyle changes, and health status change characteristics (blood pressure, blood glucose, triglycerides, HDL, and waist circumference).

## 4. Discussion

This study compared those who maintained eligibility for medical aid with those who changed from being medical aid recipients to NHI enrollees to analyze changes in health behaviors, health status, and medical expenditure depending on whether medical aid coverage was maintained.

The first significant finding was that the “changed” group showed a higher percentage of positive changes in healthy lifestyle than the “maintained” group; in the “changed group,” more people stopped smoking, fewer people newly began smoking, and more went from binge drinkers to moderate drinkers. This can be explained by the fact that, as the type of health coverage changed from medical aid with low co-payments to health insurance with increased co-payments, the “changed” group engaged in higher preventive efforts to take care of their health. This finding is in line with previous studies comparing public and private insurance in the U.S., China, Mexico, and Ghana, which found that public insurance had a significant negative effect on the self-protective behavior of the insured. In those studies, health insurance coverage was found to increase the likelihood of excessive smoking, lack of exercise, and obesity [19,36,37,38]. A healthy lifestyle maintained through self-protective behaviors such as not smoking, drinking moderately, and exercising can reduce the chances of falling ill. However, having medical aid coverage reduces the need for these self-protective behaviors by lowering the price of health care that people need to pay out of their own pocket. As they maintain a healthy lifestyle, they become healthier and use fewer healthcare resources [27,39].

The second significant outcome is the change in the subjects’ metabolic syndrome-related health status. Although the metabolic syndrome risk factors themselves were worse in the “changed” group than in the “maintained” group, the “maintained” group became worse and the “changed” group improved. From 2012 to 2014, the “maintained” group had statistically significant increases in blood glucose and waist circumference, while the “changed” group had statistically significant decreases in systolic blood pressure and triglycerides. Although the average age and the prevalence of metabolic syndrome were higher in the “changed” group to begin with, the prevalence of metabolic syndrome in the “maintained” group increased by 4.6% from 22.9% in 2012 to 27.5% in 2014, compared to 0.3%p from 29.5% in 2012 to 29.8% in 2014 in the “changed” group, indicating an increase in the prevalence of metabolic syndrome and worsening health status among the “maintained” group. This can be explained by the worsening of health in the “maintained” group. In the “changed” group, the proportion of subjects with a healthy lifestyle increased despite the worse initial health status, and the degree of deterioration regarding health status decreased compared to the “maintained” group. However, in the “maintained” group, the proportion of people with a healthy lifestyle decreased despite having better initial health status, and the degree of deterioration regarding health status was more severe compared to the “changed” group. Low socio-economic status, including income level, education level, and occupation type, was associated with an increased risk of metabolic syndrome. Since medical aid recipients with a low income are more likely to have low socio-economic status, attention should be paid to their metabolic syndrome in particular. As metabolic syndrome is an important risk factor for cardiovascular disease, if the metabolic syndrome of medical aid recipients is not managed, their health status will deteriorate further, and the burden of society to pay for their medical expenses will increase [40].

More importantly, the annual total medical expenses of the “maintained” group are higher than that of the “changed” group, with a statistically significant increase in medical expenses in the “maintained” group. Furthermore, multiple regression analyses accounting for demographic characteristics, changes in healthy lifestyle factors, and changes in health status show that maintaining medical aid alone contributes to an increase in total medical expenses. Total medical expenditure for medical aid recipients steadily increased, with per capita medical expenditures thrice that of NHI enrollees [9]. The high cost of medical care for medical aid recipients has been an ongoing issue in many countries, including South Korea [16]. Due to extremely low co-payments, there can be a moral hazard that leads to excessive healthcare utilization. In Oregon’s Health Insurance Experiment, Medicaid coverage increased emergency room visits by more than 40%, which was estimated to increase annual healthcare spending by approximately USD 120 per person [17]. In particular, the analysis also found that Medicaid significantly increased visits in all categories, except urgent and unpreventable visits. Other South Korean studies have also suggested that moral hazards for medical aid recipients, such as the change from NHI to medical aid, significantly increases the number of outpatient visits without a significant change in co-payment spending on healthcare utilization [9,24]. Therefore, an in-depth discussion regarding the appropriateness of current cost-sharing levels for medical aid recipients is warranted [9]. Additionally, the excessive utilization of healthcare services by medical aid recipients can lead to increased healthcare costs and shortages of services; therefore, medical aid needs to provide accurate assessment and support procedures, and healthcare packages need to be prioritized to ensure that recipients receive the healthcare services they need [1]. The failure to identify and provide appropriate services to people on medical aid has resulted in the excessive and unnecessary use of healthcare services and facilities. To prevent this, improving medical aid recipients’ access to preventive and primary care and improving their health is an important consideration, as this will reduce the need for the treatment of serious illnesses and emergency care [17].

The findings show that it is more common to see the ex ante moral hazard of neglecting to engage in a healthy lifestyle to improve one’s health and the ex post moral hazard of excessive consumption of healthcare services in the “maintained” group than in the “changed” group. As the majority of medical expenditure by medical aid recipients is funded through general taxes, establishing mechanisms is important to prevent the unnecessary use of healthcare services. In other words, since sharing the burden of medical expenses through appropriate co-payments is necessary, continuous research is needed to determine the appropriate level of cost sharing. However, considering that cost-sharing may limit access to health care for those who truly need it is also important [41]. Additionally, whether healthy lifestyle choices always reduce an individual’s use of healthcare services is not clear, as previous studies have shown that people with a healthy lifestyle also use more preventive health services, and their use of outpatient services does not reduce by much [42]. However, because the cost of inpatient and emergency services is much higher than that of outpatient services, adequate outpatient service utilization can reduce the use of reactive care services, which can result in higher healthcare costs later on. Therefore, educating recipients through multiple programs and encouraging them to lead a healthy life is necessary.

This study has several limitations that should be acknowledged. First, it used total medical expenses without specifically categorizing the medical services used, such as inpatient, outpatient, emergency, etc. Second, total medical expenses are influenced by other health factors and the disease characteristics of subjects. However, this study considered the total amount of medical expenses without classifying them into healthy lifestyle-related and metabolic syndrome-related expenses. Third, health status was considered only in terms of metabolic syndrome-related factors, and other health-level factors and disease severity were not considered. However, considering that the study sample regularly received national health screenings, they were considered as having a non-serious health status that allows them to voluntarily participate in health screening while leading their daily lives without experiencing serious illness or long-term hospitalization. Thus, metabolic syndrome risk factors, such as blood pressure, blood glucose, cholesterol, and obesity, were considered appropriate for representing the general health status of the sample. Fourth, as this study used data from the National Health Screenings Survey, various healthy lifestyle-related variables, such as diet, sleep, and medication abuse, may have had unmeasured cofounders. Fifth, the present study did not include screening results after 2014, warranting additional research through longer-term follow-up. Sixth, since this study was a retrospective study based on existing National Health Screening Survey data and not a prospective cohort study, gaining access to the data of subjects who did not undergo National Health Screening in 2012 and 2014 was not possible, and the control was insufficient. Finally, compared to the prevalence of metabolic syndrome in the U.S. and Korea, which exceeded 30% [28,40], in this study, only 25% of the subjects experienced metabolic syndrome. Hence, the external validity of the study is low, and the interpretation of the results is limited.

## 5. Conclusions

In this study, we analyzed the impact of maintaining medical aid recipients’ eligibility on their healthy lifestyle, metabolic syndrome-related health status, and use of healthcare services. Compared to those in the “changed” group, i.e., those who transitioned from being medical aid recipients to NHI enrollees, those in the “maintained” group, i.e., those who remained eligible for medical aid, were less likely to lead a healthy lifestyle and more likely to have poor metabolic syndrome-related health status. The “maintained” group also experienced a statistically significant increase in medical expenditure. The medical aid system was designed to address the problem of people not receiving adequate healthcare due to cost limitations, thereby promoting health equity. Since merely strengthening healthcare coverage may lead to moral hazards and the failure to link healthcare utilization and outcomes, changing the healthcare paradigm of medical aid recipients from providing treatment-oriented services to improving the quality of healthcare and ensuring preventive healthcare management is important. To improve the health status of medical aid recipients without incurring unnecessary costs, the proactive management of their healthy lifestyle choices is necessary. However, simply providing information or education does not translate into beneficial outcomes, and the attitudes and support provided by an individual’s family (and society in general) are crucial factors that must be emphasized to change the health statuses of medical aid recipients.

## Figures and Tables

**Table 1:** Comparison of subject demographics and metabolic syndrome risk factors as of 2012.

Div.		Total		2012–2014 Medical Aid Recipients (“Maintained”)		2012 Medical Aid->2014 NHI (“Changed”)		X^2^/t (*p*-Value)
		n	(%)	n	(%)	n	(%)	
Total		2366	(100.0)	1606	(67.9)	760	(32.1)	-
gender	Male	767	(32.4)	533	(33.2)	234	(30.8)	1.355 (0.132)
	Female	1599	(67.6)	1073	(66.8)	526	(69.2)	
age	40–49	1571	(66.4)	1221	(76.0)	350	(46.1)	208.57 (<0.001)
	50–59	567	(24.0)	280	(17.4)	287	(37.8)	
	60 or older	228	(9.6)	105	(6.5)	123	(16.2)	
	mean ± SD	47.53 ± 7.42		45.29 ± 6.94		51.63 ± 7.69		8.028 (<0.001)
healthy lifestyle								
smoking	non-smoking	1964	(83.0)	1327	(82.6)	637	(83.8)	0.516 (0.482)
	smoking	402	(17.0)	279	(17.4)	123	(16.2)	
drinking	moderate drinking	2145	(90.7)	1478	(92.0)	667	(87.8)	11.090 (0.001)
	binge drinking	221	(9.3)	128	(8.0)	93	(12.2)	
exercise	PA	659	(27.9)	433	(27.0)	226	(29.7)	1.978 (0.088)
	non-PA	1707	(72.1)	1173	(73.0)	534	(70.3)	
health status								
blood pressure	normal	1300	(54.9)	938	(58.4)	362	(47.6)	24.191 (<0.0001)
	risk	1066	(45.1)	668	(41.6)	398	(52.4)	
blood glucose	normal	1442	(60.9)	1013	(63.1)	429	(56.4)	9.523 (0.001)
	risk	924	(39.1)	593	(36.9)	331	(43.6)	
triglycerides	normal	1716	(72.5)	1193	(74.3)	523	(68.8)	7.741 (0.003)
	risk	650	(27.5)	413	(25.7)	237	(31.2)	
HDL	normal	1732	(73.2)	1161	(72.3)	571	(75.1)	2.121 (0.079)
	risk	634	(26.8)	445	(27.7)	189	(24.9)	
waist circumference	normal	1806	(76.3)	1230	(76.6)	576	(75.8)	0.182 (0.353)
	risk	560	(23.7)	376	(23.4)	184	(24.2)	
metabolic syndrome No. of risk factors	0	558	(23.6)	404	(25.2)	154	(20.3)	16.240 (0.006)
	1	668	(28.2)	460	(28.6)	208	(27.4)	
	2	548	(23.2)	374	(23.3)	174	(22.9)	
	3	351	(14.8)	215	(13.4)	136	(17.9)	
	4	188	(7.9)	123	(7.7)	65	(8.6)	
	5	53	(2.2)	30	(1.9)	23	(3.0)	
metabolic syndrome	No	1774	(75.0)	1238	(77.1)	536	(70.5)	11.832 (<0.001)
	Yes	592	(25.0)	368	(22.9)	224	(29.5)	

**Table 2:** Changes in healthy lifestyle factors and metabolic syndrome-related health status.

Div.	2012 → 2014	Total		“Maintained” Group		“Changed” Group		X^2^ (*p*-Value)
		n	(%)	n	(%)	n	(%)	
healthy lifestyle								
smoking	non-smoking → non-smoking	1903	(80.4)	1273	(79.5)	626	(82.4)	8.033 (0.045)
	smoking → non-smoking	74	(3.1)	47	(2.9)	27	(3.6)	
	non-smoking → smoking	61	(2.6)	50	(3.1)	11	(1.4)	
	smoking → smoking	328	(13.9)	232	(14.5)	96	(12.6)	
drinking	moderate → moderate	2038	(86.1)	1406	(87.5)	632	(83.2)	15.636 (0.001)
	binge → moderate	111	(4.7)	57	(3.5)	54	(7.1)	
	moderate → binge	107	(4.5)	72	(4.5)	35	(4.6)	
	binge → binge	110	(4.6)	71	(4.4)	39	(5.1)	
exercise	PA * → PA	294	(12.4)	197	(12.3)	97	(12.8)	2.470 (0.481)
	non-PA → PA	370	(15.6)	252	(15.7)	118	(15.5)	
	PA → non-PA	365	(15.4)	236	(14.7)	129	(17.0)	
	non-PA → non-PA	1337	(56.5)	921	(57.3)	416	(54.7)	
metabolic syndrome-related health status								
blood pressure	normal → normal	1032	(43.6)	757	(47.1)	275	(36.2)	32.112 (<0.001)
	risk → normal	260	(11.0)	177	(11.0)	83	(10.9)	
	normal → risk	268	(11.3)	181	(11.3)	87	(11.4)	
	risk → risk	806	(34.1)	491	(30.6)	315	(41.4)	
blood glucose	normal → normal	1086	(45.9)	769	(47.9)	317	(41.7)	11.683 (0.009)
	risk → normal	265	(11.2)	178	(11.1)	87	(11.4)	
	normal → risk	356	(15.0)	244	(15.2)	112	(14.7)	
	risk → risk	659	(27.9)	415	(25.8)	244	(32.1)	
triglycerides	normal → normal	1448	(61.2)	1012	(63.0)	436	(57.4)	13.765 (0.003)
	risk → normal	275	(11.6)	161	(10.0)	114	(15.0)	
	normal → risk	268	(11.3)	181	(11.3)	87	(11.4)	
	risk → risk	375	(15.8)	252	(15.7)	123	(16.2)	
HDL	normal → normal	1421	(60.1)	949	(59.1)	472	(62.1)	4.108 (0.250)
	risk → normal	266	(11.2)	179	(11.1)	87	(11.4)	
	normal → risk	311	(13.1)	212	(13.2)	99	(13.0)	
	risk → risk	368	(15.6)	266	(16.6)	102	(13.4)	
waist circumference	normal → normal	1602	(67.7)	1088	(67.7)	514	(67.6)	1.890 (0.596)
	risk → normal	167	(7.1)	106	(6.6)	61	(8.0)	
	normal → risk	204	(8.6)	142	(8.8)	62	(8.2)	
	risk → risk	393	(16.6)	270	(16.8)	123	(16.2)	
metabolic syndrome	No → No	1515	(64.0)	1066	(66.4)	449	(59.1)	16.047 (0.001)
	Yes → No	199	(8.4)	115	(7.2)	84	(11.1)	
	No → Yes	259	(10.9)	172	(10.7)	87	(11.4)	
	Yes → Yes	393	(16.6)	253	(15.8)	140	(18.4)	

* PA: physical activity.

**Table 3:** Differences in metabolic syndrome risk factors and medical expenses.

Div.		“Maintained” Group	“Changed” Group	t (*p*-Value)
		M ± SD	M ± SD	
Systolic blood pressure (mmHg)	2012	118.81 ± 14.83	122.90 ± 15.07	−6.228 (<0.001)
	2014	119.31 ± 14.65	121.70 ± 14.60	−3.706 (<0.001)
	Difference	0.51 ± 14.85	−1.19 ± 15.29	2.576 (0.010)
	t (*p*)	−1.366 (0.172)	2.154 (0.032)	-
Diastolic blood pressure (mmHg)	2012	74.99 ± 10.38	76.81 ± 10.11	−4.026 (<0.001)
	2014	74.65 ± 9.88	76.06 ± 10.02	−3.234 (0.001)
	Difference	−0.34 ± 10.50	−0.75 ± 11.07	0.858 (0.391)
	t (*p*)	1.302 (0.193)	1.875 (0.061)	-
blood glucose (mg/dL)	2012	98.88 ± 27.19	102.53 ± 27.40	−3.046 (0.002)
	2014	101.30 ± 32.86	103.36 ± 29.20	−1.474 (0.141)
	Difference	2.42 ± 27.72	0.82 ± 27.97	1.304 (0.192)
	t (*p*)	−3.495 (<0.001)	−0.809 (0.419)	-
triglycerides (mg/dL)	2012	130.46 ± 101.41	135.20 ± 94.27	−1.085 (0.278)
	2014	130.78 ± 97.31	128.64 ± 87.00	0.516 (0.606)
	Difference	0.32 ± 96.35	−6.56 ± 86.60	1.673 (0.094)
	t (*p*)	−0.132 (0.895)	2.088 (0.037)	-
HDL (mg/dL)	2012	54.58 ± 21.32	56.17 ±25.18	−1.599 (0.110)
	2014	54.49 ± 19.50	55.22 ± 13.34	−0.941 (0.347)
	Difference	−0.09 ± 24.02	−0.95 ± 24.81	0.802 (0.422)
	t (*p*)	0.150 (0.881)	1.053 (0.293)	-
waist circumference (cm)	2012	79.54 ± 10.17	80.41 ± 9.44	−2.003 (0.045)
	2014	80.16 ± 10.27	80.55 ± 9.38	−0.884 (0.377)
	Difference	0.62 ± 6.21	0.14 ± 5.98	1.796 (0.073)
	t (*p*)	−4.028 (<0.001)	−0.643 (0.521)	-
total medical expenses (USD)	2012	1914.20 ± 2851.05	1446.77 ± 2215.20	3.986 (<0.001)
	2014	2103.46 ± 3152.64	1550.36 ± 2546.46	4.228 (<0.001)
	Difference	189.26 ± 2499.99	103.59 ± 2428.89	0.785 (0.432)
	t (*p*)	−3.034 (0.002)	−1.176 (0.240)	-

(1 USD = 1300 KRW).

**Table 4:** How changes in medical aid eligibility can increase total medical expenses.

Variable	Level	Model 1 *			Model 2 **			Model 3 ***		
		OR	95% CI	*p*-Value	OR	95% CI	*p*-Value	OR	95% CI	*p*-Value
medical aid eligibility	Changed	1.000			1.000			1.000		
	Maintained	1.368	1.141–1.641	<0.001	1.400	1.165–1.683	<0.001	1.421	1.180–1.710	<0.001
Fit Statistics	AIC ****	3251.992			3256.328			3269.221		
	C-statistics	0.543			0.565			0.579		

* Adjustment variable of Model 1: demographic characteristics (gender and age), ** Adjustment variable of Model 2: demographic characteristics (gender and age) and healthy lifestyle change characteristics (smoking, drinking, and exercise) *** Adjustment variable of Model 3: demographic characteristics (gender and age), healthy lifestyle change characteristics (smoking, drinking, and exercise), and health status change characteristics (blood pressure, blood glucose, triglycerides, HDL, and waist circumference), **** AIC: Akaike Information Criterion.

## Data Availability

Research data are available upon request. To request the data, contact the corresponding author of the article.
